# Teleconnection between the Asian Polar Vortex and surface PM_2.5_ in China

**DOI:** 10.1038/s41598-020-76414-6

**Published:** 2020-11-10

**Authors:** Lihua Zhou, Jing Zhang, Xiaohui Zheng, Siguang Zhu, Yueming Hu

**Affiliations:** 1 College of Global Change and Earth System Science, Beijing Normal University, Beijing, 100875 China; 2 China Meteorological Administration Training Center, CMA, Beijing, 100081 China; 3 Collaborative Innovation Center on Forecast and Evaluation of Meteorological Disasters/Key Laboratory of Meteorological Disaster of Ministry of Education, Nanjing University of Information Science and Technology, Nanjing, 210044 China; 4 China Meteorological Administration Public Meteorological Service Center, CMA, Beijing, 100081 China

**Keywords:** Environmental sciences, Climate sciences, Atmospheric science

## Abstract

Atmospheric fine particulate matter (PM_2.5_) pollutions are of particular concern because of their direct and indirect harm to humans and organisms. China has suffered from severe air pollution for the past ten years, related to heavy pollution emissions and compounded by the effects of atmospheric circulation. This study applied statistical methods, observational data of ground pollutants, and meteorological data to analyze the impact of large-scale atmospheric circulations on PM_2.5_ pollution over China. Empirical orthogonal function (EOF) analysis was used to evaluate the main PM_2.5_ patterns and total contributions of the leading four EOFs. The results indicate that the total contributions of the leading four EOFs accounted for 50.5% of the total variance, reflecting four main types of PM_2.5_ pollution, namely, overall pollution phase, north–south phase, east–west phase and north–center–south phase, with contributions of 28.4%, 9.7%, 6.5% and 5.9%, respectively. We selected indices of the Asian Polar Vortex (APV) to analyze the impact of large-scale atmospheric circulations on PM_2.5_ pollution over China. The most pronounced APV control occurred in Beijing and its surroundings, specifically, along the Bohai Sea and the Northeast Plain.

## Introduction

Atmospheric fine particulate matters (PM_2.5_) are important components of atmospheric aerosols, although its effects are highly variable and uncertain. It has major impacts on human health^1^, radiative forcing^2^ and climate change^3,4^. In practice, PM_2.5_ levels are an important indicator of air pollution. Although early studies were mostly based on simulations and satellite data inversion^5,6^, the increase in observation sites in recent years provides a strong foundation to study PM_2.5_ pollution patterns more accurately.

PM_2.5_ pollution is extremely serious in China, concerning the government and researchers for nearly a decade. The spatial distribution of PM_2.5_ over continental China is highly variable. Typically, three strongly polluted regions are identified: the North China Plain (NCP) (including Beijing–Tianjin–Hebei), the Yangtze River Delta (YRD) and the Pearl River Delta (PRD). Additionally, the Guanzhong Plain (GZP) and the Sichuan Basin (SCB) have major amounts of PM_2.5_ pollution^7^.

The above studies were mostly based on analyses of time series data obtained from site observations carried out to determine haze compositions. The simulation of aerosol composition is also a common study method. Given that the scale of a weather system is often large and that even a mesoscale system can reach several hundred kilometers in size, it has not been easy to measure the overall impact of weather systems on the distribution of PM_2.5_ pollution, thereby making it equally unfeasible to assess the impact of climate change on PM_2.5_. To address this issue, we selected the entire region of China as the research space to study over a 50-month time span. We focused on large temporal and spatial scales instead of selecting a province or a city region. Given the increase of air quality monitoring sites established in China, the spatiotemporal analysis of haze can now be undertaken more comprehensively. Likewise, there is a greater database of local meteorological variables available for analysis. One advantage of these new data is that we can now analyze data from small to large spatial scales more effectively.

Already, studies have shown that daily variation in meteorology can explain up to 50% of the daily PM_2.5_ variability in the US^8^. This indicates a potential impact of synoptic change on PM_2.5_ pollution. In addition to emission sources, climate change also affects air pollution^9^. Another aspect of climate change that needs to be considered is its effect on atmospheric chemical processes. For example, an increase in air temperature in the Arctic in winter will increase atmospheric oxidation over land, resulting in more ${\mathrm{SO}}_{2}$ being converted to ${\mathrm{SO}}_{4}^{2-}$. This is important because sulfate is one of the main components of haze contributing to air pollution^10,11^. Simulations of the impacts of predicted global climate change on air quality indicate that it will increase summertime surface ozone (O_3_) in polluted regions by 1–10 ppb, with the greatest impact on the O_3_ in urban areas; however, this change will also affect PM concentrations in polluted environments by 0.1–1 mg/m^3^ over the coming decades^9^. To address large-scale weather patterns, we chose not to select local specific meteorological parameters, such as temperature, humidity, precipitation, synoptic pressure, and wind, as our research object^12^, but rather one that reflected the large circulation system that controls weather and climate in China, i.e., the polar vortex (PV). Enhanced Arctic warming relative to mid-latitudes may cause more persistent weather patterns in mid-latitudes, with higher incidences of extreme weather^13^. Because the PV is affected by Arctic sea ice volumes via land-air interactions, its values reflect variation in Arctic sea ice and related global environmental changes. The East Asian winter monsoon is strongly influenced by the winter Arctic Oscillation. When this oscillation is in its positive phase, the monsoon is weaker than average, and air temperatures from near the surface to the middle troposphere are approximately 0.5–2 °C higher than average along the East Asian coast, including the coast of East China. Clearly, the East Asian winter monsoon is the main factor affecting winter weather in China^14^. However, the Asian Polar Vortex (APV) indirectly affects the distribution and level of PM_2.5_ in China by controlling its regional synoptic systems^15,16^. In addition, the change in APV is greatly affected by Arctic sea ice volumes. Therefore, anomalous Arctic sea ice will also affect PM_2.5_ pollution distributions in China on a climatic scale^17^. Thus, the APV reflects the impact of global climate change on air quality^18^. The PV is a large low-pressure system that is entrenched in the Arctic. The part of the PV located over Asia is called the Asian PV (APV). It is an important factor affecting climate and weather in China. This large-scale circulation system affects wide-ranging distributions of local meteorological parameters in China, including temperature, humidity and precipitation^19^, which in turn affect the formation, development, sedimentation and transmission of haze^20,21^. Therefore, the APV is an important indirect factor affecting PM_2.5_. It likely determines the frequency and distribution of PM_2.5_ pollution in China by affecting weather systems over China. Analysis of the relationship between circulation patterns and air quality during constant emission periods suggests that atmospheric circulation types are the primary drivers of day-to-day variations in pollutant concentrations^22–24^.

The most common method of spatiotemporal decomposition in the field of geosciences is the empirical orthogonal function (EOF)^25^. Compared with other functions that simply analyze the spatiotemporal distribution, the EOF method performs spatiotemporal separation and extracts the main distribution modes. We used this method for daily and monthly analyses and selected the typical distribution of pollution to reverse the distribution of atmospheric circulation^26^. To link the spatial distribution of pollution in China to the APV, we used a teleconnection method^18^. This method is used to study the atmosphere on the planetary scale. Atmospheric teleconnection refers to a high correlation between climatic factors in two places, separated by thousands of kilometers^27,28^. Observations also show that an anomaly of circulation in one region can cause anomalies in circulation in another distant region^29^. The teleconnection method is widely used not only in atmospheric circulation but also in dust research^30,31^. In this study, we applied this method to study PM_2.5_. Because the focus of this paper deals with large spatial scales, we do not discuss PM_2.5_ effects related to local emissions^32^. The spatial distribution of regional emissions is relatively stable and is not discussed in this study, and furthermore, it is not the best way to distinguish the small changes in emissions by the statistical analysis method. However, the spatial distribution of weather changes greatly with time. Compared to emissions, this is a more significant factor affecting PM_2.5_ distribution.

## Results

### General description of distributions and seasonal changes of PM_2.5_

**Figure 1:**
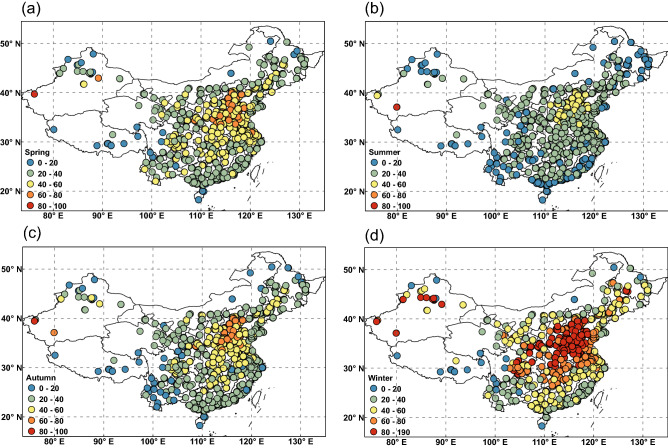
Spatial distributions of seasonal PM_2.5_ (unit: μg m^3^) patterns in (**a**) spring, (**b**) summer, (**c**) autumn, and (**d**) winter for January 2015 to February 2019. PM_2.5_, fine particulate matter. The maps were created by software ArcGIS 10.2.2(https://www.arcgis.com).

**Figure 2:**
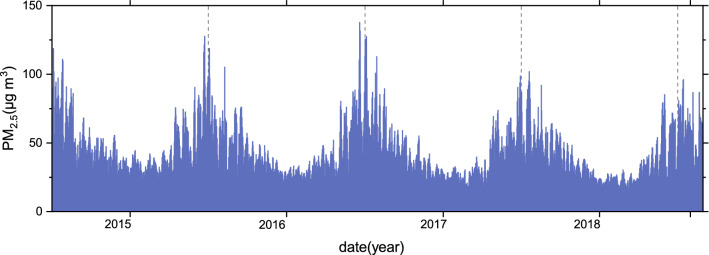
Time series of PM_2.5_ (unit: μg m^3^) over China. The figure is created by Origin 2018 software (https://www.originlab.com/).

The seasonal mean PM_2.5_ concentration fields based on data from all Chinese observation sites during 2015–2019 are shown in Fig. 1, and the time series of PM_2.5_ over China is shown in Fig. 2. The NCP was characterized by the highest level of PM_2.5_ throughout the year. Winter was clearly the most polluted season (Fig. 1d), with the least pollution occurring in summer (Fig. 1b) and transitional values in spring and autumn (Fig. 1a,c). In China, the mean value of PM_2.5_ was 63 μg/m^3^ in winter; in addition, there are 375 sites with an average concentration value exceeding 75 μg/m^3^. Figure 1b displays a summer distribution of PM_2.5_. The mean value of PM_2.5_ was 28 μg/m^3^ in summer, and there are only 3 sites with an average concentration value exceeding 75 μg/m^3^. These high-value PM_2.5_ locations are located in North China (NCP). The variability of the PM_2.5_ time series in most parts of China is relatively large, especially in the heavily polluted areas of North China (Jing-Jin-Ji). Because the density of observation sites in the east is much higher than in the west, we mainly discuss the PM_2.5_ distribution patterns of central and eastern parts of China.

### Empirical orthogonal function analysis of spatial distributions and temporal evolutions of PM_2.5_

We deseasonalized the original data by subtracting 31-day moving averages before the EOF analysis. The EOF method is one of the principal component analysis methods. The method introduction section introduces the algorithm. To calculate the variance contribution rate, the variance of matrix $\mathbf{X}$ is represented by the measurement of the eigenvalue. The larger the λ, the more important the corresponding EOF and the greater its contribution to the total variance. The contribution of the k_th_ pattern to the total variance is: $\frac{{\lambda }_{k}}{{\sum }_{i=1}^{m}{\lambda }_{i}}\times 100{\%}$. The error ranges of the leading four eigenvalues did not overlap, and all four of them passed the significance test. The cumulative variance contribution rate of the leading four eigenvectors reached 50.5%, although the cumulative variance contribution rate of the leading two leading eigenvectors was close to 38%. This suggests that these two eigenvalues explain both spatial and temporal distribution patterns of PM_2.5_ over China from 2015 to 2019.

**Figure 3:**
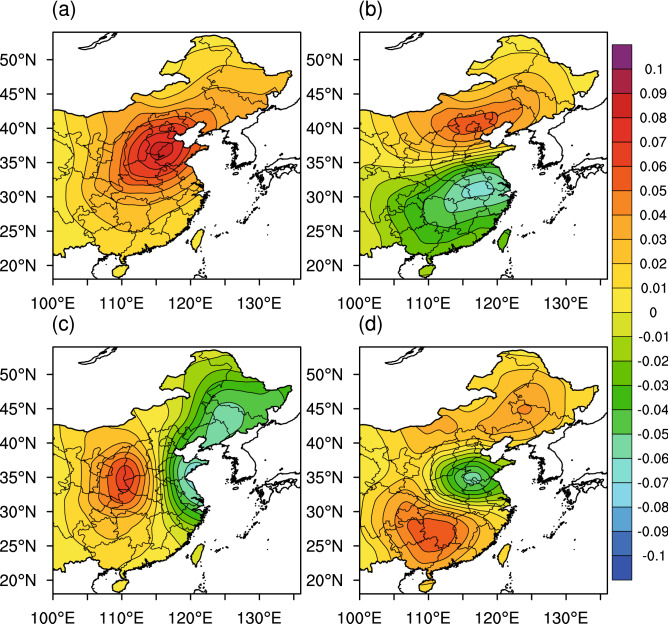
Spatial distribution patterns of the leading four PM_2.5_ mass concentration eigenvectors in China: (**a**) EOF-1, (**b**) EOF-2, (**c**) EOF-3 and (**d**) EOF-4. PM_2.5_, fine particulate matter; EOF, empirical orthogonal function. The original data are detrended and deseasonalized by subtracting 31-day moving averages before the EOF analysis. The maps were created by NCL6.3.0 software (https://www.ncl.ucar.edu/).

**Figure 4:**
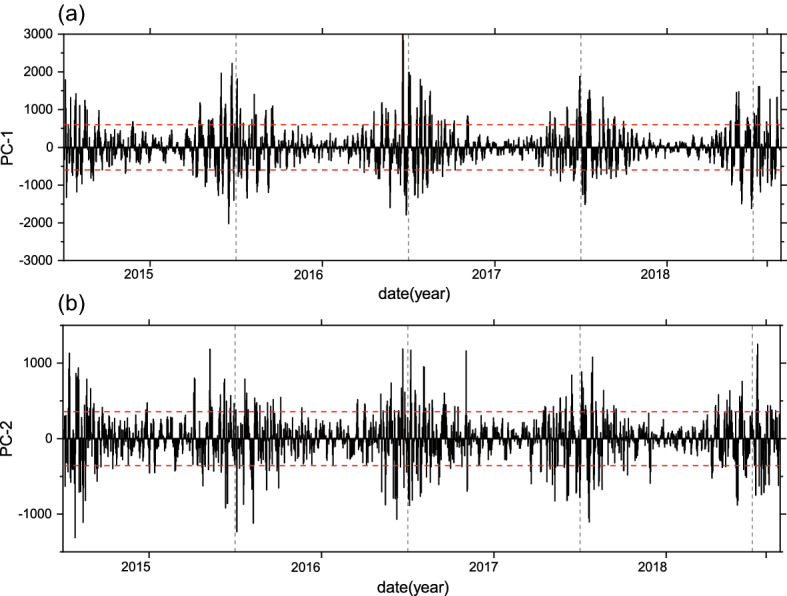
(**a**) Time series of PC-1 corresponding to EOF‐1, outside of the two red dotted lines are the typical periods (PC-1>$1.28\upsigma$ and PC-1 < − $1.28\upsigma$ process); (**b**) time series of PC-2 corresponding to EOF‐2, outside of the two red dotted lines are the typical periods (PC-2>$1.28\upsigma$ and PC-2 < − $1.28\upsigma$ process), PC represents the importance of the corresponding EOF. The maps were created by Origin 2018 software (https://www.originlab.com/).

The variance contribution rate of the EOF-1 eigenvector was 28.4%, which was much higher than the contribution rate of all other EOFs. It determines the main spatial distribution pattern of PM_2.5_ in China. Figure 3a shows that the eigenvalues of almost all stations in EOF-1 are positive, indicating that the trend of PM_2.5_ changes is consistent over the entire country. Moreover, this distribution is closely related to the emissions inventory compiled by Tsinghua University^33,34^, indicating that emissions dominate the local cumulative pollution. A high value center occurred in Central China, reflecting the high concentration of PM_2.5_ in this area. The PM_2.5_ throughout the central region is much higher than other regions. In contrast, the variance contribution rate of the EOF-2 eigenvector is 9.7% but also clearly reflects the regional PM_2.5_ spatial distribution. Figure 3b shows the characteristics of PM_2.5_ pollution of EOF-2. The distribution pattern is defined as the north–south phase, formed by the Qinling Mountains and Huaihe River, with a positive zone to the north and a negative zone to the south. The positive center is in the Beijing–Tianjin–Hebei region, while the negative center is in the Yangtze River Basin. This indicates that when there is an increase in PM_2.5_ in the Beijing–Tianjin–Hebei region, there is a corresponding decrease in PM_2.5_ in the southern region, and vice versa. Figure 3c shows that their corresponding distribution pattern is the east–west phase, with a positive zone in the central region and a negative zone in the northeast and eastern coastal areas. Its positive center is in the Guanzhong region, while the negative center is in the Northeast Plain. This indicates that an increase in PM_2.5_ in the central region is matched by a decrease in PM_2.5_ in the northeast region, and vice versa. Figure 3d shows that this distribution pattern is the north–central–south phase and characterized by a decrease in PM_2.5_ in the central to eastern regions and an increase in PM_2.5_ in other regions. PC is the time factor of EOF and represents the importance of the corresponding EOF mode. The larger the absolute value of PC, the more significant the EOF pattern during this period (Fig. 4a,b). Therefore, we can discover the time when the two dominant patterns occur according to the value of PC.

### Correspondence of Asian atmosphere circulation and extreme PM_2.5_ pollution

**Figure 5:**
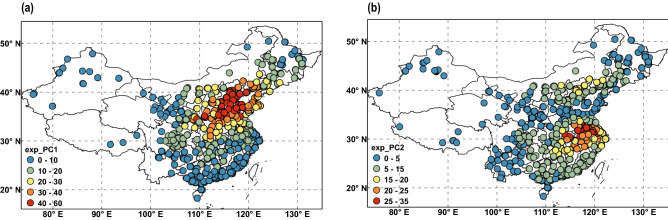
Percentage of PM_2.5_ variance explained by the two leading principal components (PCs): (**a**) PC-1; and (**b**) PC-2. PM_2.5_, fine particulate matter. The maps were created by ArcGIS 10.2.2 software (https://www.arcgis.com).

The EOFs are eigenfunctions of the data set’s covariance matrix, whose eigenvalues equal the variances of the corresponding PCs. The principal component (PC) corresponds to the time series variation, which reflects the weight change of the corresponding EOF pattern over time. We used the direct correlation between PCs and PM_2.5_ to evaluate the contribution of the leading PCs to the total PM_2.5_ variance. The original values of PM_2.5_ at each site were detrended and deseasonalized by subtracting 31-day moving averages before calculating the correlation. The percentage contributions of the two leading PCs of PM_2.5_ were determined as the square (× 100%) of the correlation coefficients between the PM_2.5_ time series at each site and the two leading PCs of the PM_2.5_ field. This percentage was considered invalid if the site did not pass the significance test. The resulting percentages are displayed in Fig. 5. The most pronounced regions controlled by EOF-1 were in the central and northern regions (Northeast Plain, NCP, GZP). There were 270 sites with percentage contributions of PM_2.5_ variance explained by EOF-1 over 30% and 64 sites with percentage contributions over 50% (Fig. 5a). In contrast, the most pronounced regions controlled by EOF-2 were in Beijing and surrounding areas and the Yangtze River Plain. There were 198 sites with percentage contributions of PM_2.5_ variance explained by EOF-2 over 20% and 20 sites with percentage contributions over 30% (Fig. 5b).

**Figure 6:**
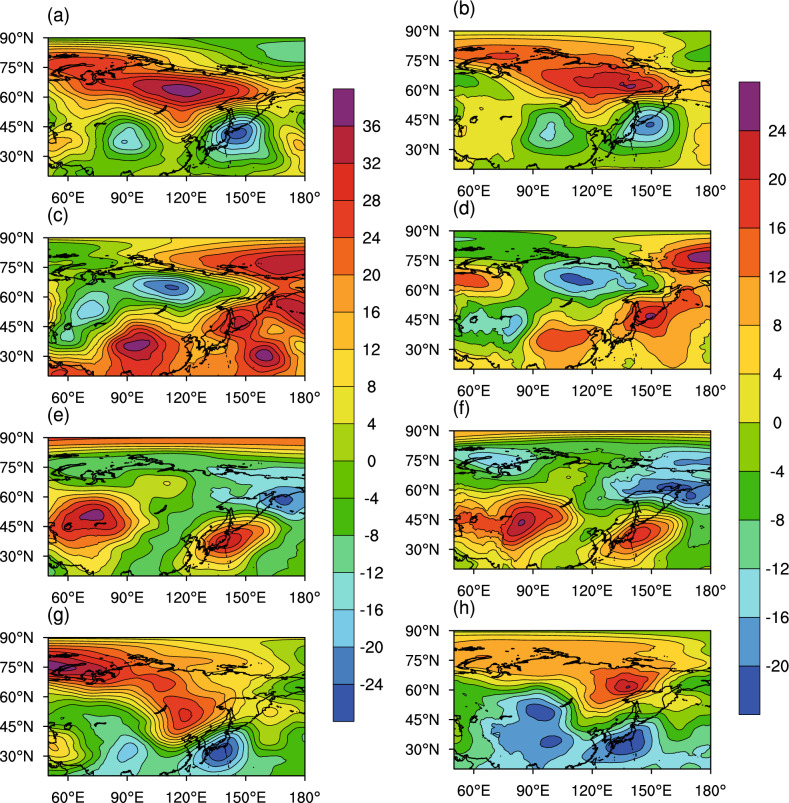
Atmospheric circulation geopotential height (unit: gpm) anomaly in the case of PC-1 > 1.28σ, corresponding to (**a**) 500-hPa and (**b**) 850-hPa fields; in the case of PC-1 <  − 1.28σ, corresponding to (**c**) 500-hPa and (**d**) 850-hPa fields; in the case of PC-2 > 1.28σ, corresponding to (**e**) 500-hPa and (**f**) 850-hPa fields; (**g**) 500-hPa and (**h**) 850-hPa (Unit: km). PCs are the leading principal components of PM_2.5_. The maps were created by NCL6.3.0 software (https://www.ncl.ucar.edu/).

The extreme PC value indicates that the EOF pattern was the dominant PM_2.5_ distribution at that time during the study period. Therefore, we selected atmospheric circulation fields corresponding to EOF-1 and EOF-2 for meteorological background analysis according to the values of PC-1 and PC-2, respectively. Given the spatial characteristics of PM_2.5_ described above, we focused on spatial distributions consistent with a mesoscale weather system of a few hundred kilometers. We chose typical PM_2.5_ distributions based on the PCs because PCs represent the weight of EOFs. We chose the case with the PC value belonging to the highest 10% (PC > 1.28σ case) or the lowest 10% (PC < − 1.28σ case during the study period, which corresponds to the typical distribution of PM_2.5_; at the same time we determined the corresponding characteristics of the atmospheric circulation field (500 hPa and 850 hPa) during this period, as shown in Fig. 6. The extremum of the PC shows the time when a typical PM_2.5_ distribution occurred and the simultaneous atmospheric circulation fields were found, such that the two are linked.

Based on EOF spatial distribution characteristics, the two leading PCs embody four pollution patterns: Type (1) the highest pollution in North China and high pollution over the entire country ($\mathrm{PC}-1>1.28\sigma \mathrm{ case}$);Type (2) low pollution in North China and over the entire country ($\mathrm{PC}-1<-1.28\sigma \mathrm{ case}$); Type (3) concentrated pollution in the Beijing–Tianjin–Hebei region and its surrounding areas ($\mathrm{PC}-2>1.28\sigma \mathrm{ case}$); and Type (4) reduced pollution in the Beijing–Tianjin–Hebei region and North China, but elevated pollution in the south ($\mathrm{PC}-2< -1.28\sigma \mathrm{ case}$). At the same time, according to the PC value, the mean atmospheric circulation during the typical PM_2.5_ pattern is identified for analysis. According to these four types of pollution, we selected corresponding atmospheric circulation fields. The results show that the PM_2.5_ distribution pattern is related to the atmospheric circulation field.

When the pollution pattern corresponds to Type (1), the highest pollution in North China, the distribution of atmospheric circulation is shown (Fig. 6a,b). The air pressure over Siberia is higher, and the air pressure over the Western Pacific and the Tibetan Plateau is lower according to the 500 hPa and 850 hPa contour maps. When the pollution pattern corresponds to type (2), the circulation is completely different from Type (1) (Fig. 6c,d)^35^. When the pollution corresponds to type (3), with pollution concentrated in Beijing and its surrounding areas, atmospheric circulation is shown (Fig. 6e,f). There are two high pressures in western China and Japan at the same time. The pollution corresponds to Type (4), with serious pollution in the south^36^. The atmospheric circulation (related to Rossby waves) corresponding to these PM_2.5_ patterns are very different. Therefore, different PM_2.5_ distributions correspond to completely different atmospheric circulation fields. The APV seriously affects the distribution of polluted areas in China. The 500-hPa and 850-hPa geopotential height fields are used to represent the atmospheric circulation field. By observing these 500 hPa and 850 hPa potential height fields, we find that each type of PM_2.5_ pattern corresponds to a different Arctic circulation; thus, we consider the relationship between the APV and PM_2.5_ distribution.

### Percentage of PM_2.5_ variance explained by Asian Polar Vortex

PV is cyclonic circulations around the Arctic. The part of the PV located over Asia (60° E–150° E) is called the Asian PV (APV). The area index of the APV (AIAPV) and strength index of the APV (SIAPV) are often used to measure its variations. On a monthly scale weather forecasting, China weather changes can be viewed as the result of the interaction between the PV and the subtropical high circulation system. According to research by the diagnostic and prediction group of the National Climate Center of China Meteorological Administration (NCC-CMA), the southernmost boundary of the PV in the northern hemisphere can be regarded as the boundary between the two systems^37^. Therefore, it can be considered the area surrounded by the axis of the westerly belt on the 500-hPa isobaric surface. That is, the area is surrounded by the north of the geopotential height near the westerly. The area of the PV in the range of 60° E–150° E is called the Area Index of Asian Polar Vortex (AIAPV). The intensity of the PV is expressed by the air weight between the 500-hPa isobaric surface and the potential height surface where the southern geopotential height of the PV is located. The part between 60E-150E is called the Strength Index of Asian Polar Vortex (SIAPV). The AIAPV and the SIAPV are two parameters used to describe the characteristics of APV; thus, calculating the correlations between these two parameters and PM_2.5_ can reflect the correlation between APV and PM_2.5_.

Research by the Climate Center shows that the AIAPV is closely related to the weather changes in China. The AIAPV is obviously negatively correlated with winter temperature in China. In the summer, the negative correlation area is limited to the northeast and northwest, and the other areas have a positive correlation. In addition, the AIAPV has a certain relationship with the distribution of summer precipitation in China. When the polar vortex area increases, there is less precipitation in the northeast and south of the Yangtze River, and more precipitation in most areas of the Huaihe River, West China, and Northwest. Because China is located in the monsoon zone, the local weather in winter is mainly affected by APV activity; thus, the SIAPV is closely related to temperature and other meteorological factors in China.

**Figure 7:**
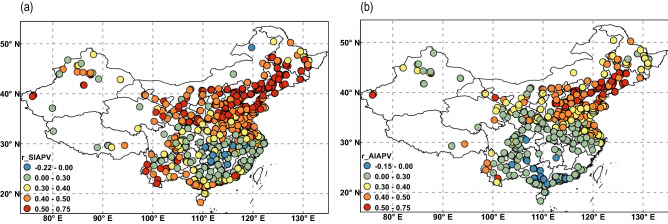
The correlation coefficients between PM_2.5_ and (**a**) SIAPV and (**b**) AIAPV. Monthly AIAPV and SIAPV data were deseasonalized by subtracting nonoverlapping 3-month averages and then normalized; monthly total PM_2.5_ were also deseasonalized in the same way^7^. PM_2.5_, fine particulate matter; AIAPV, area index of the Asian Polar Vortex; SIAPV, strength index of the Asian Polar Vortex. The maps were created by ArcGIS 10.2.2 software (https://www.arcgis.com).

**Figure 8:**
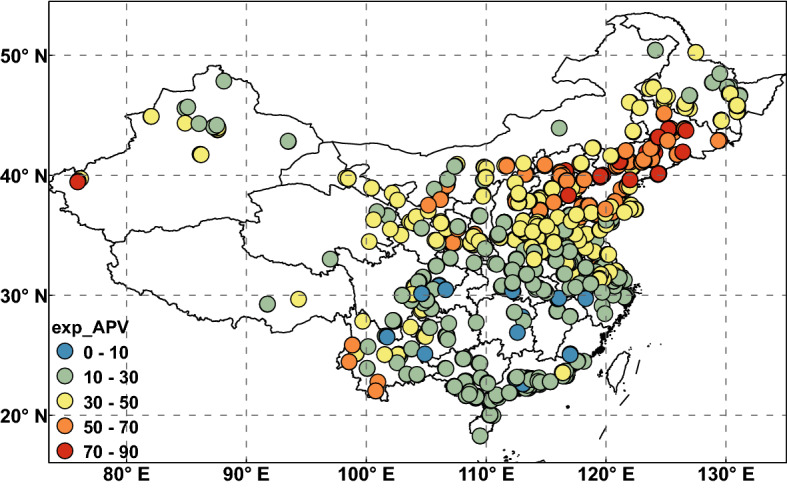
Percentage of PM_2.5_ variance explained by APV. PM_2.5_, fine particulate matter; APV, Asian Polar Vortex. The map is created by ArcGIS 10.2.2 software (https://www.arcgis.com).

There is a significant positive correlation between PM_2.5_ and the APV time series (Fig. 7). The correlation coefficient is greater than 0.5 at most sites in North and Northeast China. Moreover, the correlation coefficient between the SIAPV and PM_2.5_ (Fig. 7a) is greater than that between the AIAPV and PM_2.5_ (Fig. 7b). A higher correlation occurs in North China than in South China. To validate the APV’s synoptic control of PM_2.5_ variability and its geographical distribution, subcorrelation of its two indices was carried out. The percentage explained by APV was defined as the sum of the squares (× 100%) of the correlation coefficients between PM_2.5_ time series at each point and the AIAPV and SIAPV time series. The resulting percentages are displayed in Fig. 8. The numbers of sites where the percentage is more than 70% is 33, and the numbers of sites where the percentages are more than 30% and 50% are 569 and 206, respectively. The most pronounced APV control occurred in the north and northeast regions, with more than 50% of the PM_2.5_ variance explained by the APV. This suggests that PM_2.5_ and APV are highly correlated in northern China. More than 70% of PM_2.5_ variance could be explained by the APV in Beijing and its surroundings. This also illustrates that Arctic atmospheric circulation mainly affects the PM_2.5_ pollution level in northern China.

## Conclusion and discussion

The focus of this study was the relationship between large atmospheric circulations and the spatial distributions of PM_2.5_ over China. The original daily data were detrended and deseasonalized by subtracting 31-day moving averages before the EOF analysis. The monthly PM_2.5_ and APV (SIAPV and AIAPV) data were deseasonalized by subtracting nonoverlapping 3-month averages and then normalized. We performed the EOF decomposition of PM_2.5_ to obtain the dominant pattern of PM_2.5_ and its weight coefficient (PC). When the weight coefficient PCs took extreme values, the corresponding significant EOFs represented the PM_2.5_ distribution of this period. At the same time, the 500-hPa and 850-hPa potential height fields from this moment were selected to describe the atmospheric circulation corresponding to this type of PM_2.5_ distribution, such that the spatial distribution of the atmospheric circulation and the PM_2.5_ occurred at the same time. By observing these 500-hPa and 850-hPa potential height fields, we found that each type of PM_2.5_ corresponds to a different Arctic circulation; thus, we considered the relationship between the APV and PM_2.5_ distribution because the definition of the SIAPV and AIAPV comes from the 500-hPa circulation. The APV was considered the most important climatic factor affecting Chinese weather, and its evolution was represented by the AIAPV and SIAPV. Finally, the distributions of APV and PM_2.5_ were linked by teleconnection. Our findings are summarized below.

PM_2.5_ levels show that North China is the most polluted region of China. The annual winter PM_2.5_ average of China is 63 µg/m^3^, and the summer PM_2.5_ average is less than 30 µg/m^3^. Winter is the most polluted season, with the least pollution in summer and transitional values in spring and autumn. There is a large variation of daily PM_2.5_. This shows that areas with serious pollution also have large daily changes.

According to the EOF statistical analysis of PM_2.5_ in China, the cumulative variance contribution of the four eigenvectors is 50.5%; therefore, they reflect the main characteristics of the PM_2.5_ space field. The EOF-1 variance contribution reached 28.4% and the variance contribution of EOF-2 reached 9.7%, respectively. Based on the leading two PCs, we selected height fields corresponding to the top 10% and bottom 10% of PCs and found that extreme levels of PM_2.5_ are closely related to the APV.

Obvious correlations were observed between the local PM_2.5_ time series and the APV time series. There were 350 sites with a correlation coefficient greater than 0.5 between PM_2.5_ and SIAPV and 126 sites with a correlation coefficient greater than 0.5 between PM_2.5_ and the AIAPV. Moreover, the correlation coefficient between the SIAPV and PM_2.5_ was greater than that between the AIAPV and PM_2.5_. A higher correlation was found in North and Northeast China. Finally, we determined the percentage effect of APV on PM_2.5_. The number of sites where the percentage is more than 70% was 33, and the number of sites where the percentages were more than 30% and 50% were 569 and 206, respectively. The most pronounced APV control occurred in the north and northeast regions. This result suggests that PM_2.5_ and APV are highly correlated in northern China. More than 70% of the PM_2.5_ variance could be explained by the APV in Beijing and its surroundings, especially along the Bohai Sea and the Northeast Plain. This shows that there is a significant relationship between the APV and PM_2.5_ in China, and this study is conducive to predicting the potential impact of climate change on PM_2.5_ pollution.

## Methods

Raw hourly average PM_2.5_ mass concentrations (hereafter PM_2.5_) were derived from the National Urban Air Quality Real-time Release Platform of Environmental Protection in China (MEPC; https://106.37.208.233:20035/)^38^. The PM_2.5_ data covered a 50-month period from January 2015 to February 2019. The daily PM_2.5_ data were processed to give a 24-h geometric mean.

Meteorological parameters, including hourly heights of 500 hPa (H_500_) and 850 hPa (H_850_), were obtained from the National Centers for Environmental Protection (NCEP) Climate Forecast System Version 2 (CFSv2) Selected Hourly Time-Series Products (0.5° × 0.5°). The H_500_ and H_850_ values were used to characterize atmospheric circulation. The daily average of each meteorological parameter was determined from hourly values. According to the definitions of the Area Index of Asian Polar Vortex (AIAPV) and the Strength Index of Asian Polar Vortex (SIAPV), these are closely related to the 500-hPa circulation. Climatic factors, including the area index of the APV (AIAPV) and strength index of the APV (SIAPV), are given in the regular climate data released by the Climate Center of China Meteorological Administration. EOF^39^, also known as eigenvector analysis or principal component analysis, is a method used to analyze the structural characteristics of matrix data and extract the main eigenvectors of the raw data. This methodology is widely used in geosciences and atmospheric science. It has also been used to study air pollution in recent years^40,41^. In this study, we only performed EOF decomposition on PM_2.5_. We can find the leading PM_2.5_ pattern and the time when the significant leading PM_2.5_ pattern appears by EOF decomposition on PM_2.5_, and then we select 500 hPa and 850 hPa in this case for analysis and comparison. The original PM_2.5_ data are detrended and deseasonalized by subtracting 31-day moving averages before the EOF analysis.

EOF decomposition is a spatiotemporal decomposition, which resolves the spatial structure of the actual field by decomposing the raw matrix into a spatial function and a temporal function^42^. The spatial function summarizes the geographical distribution characteristics of the field; it is composed of a linear combination of spatial point variables of the field. Therefore, the eigenvectors correspond to spatial samples, are also called spatial eigenvectors or spatial modalities (EOFs), which reflect the spatial distribution characteristics of the element field. The principal component (PC) corresponds to the time series variation, which reflects the weight change of the corresponding EOF pattern over time. Therefore, EOF analysis is a spatiotemporal decomposition, namely:

$$\mathbf{X}=\mathbf{E}\mathbf{O}\mathbf{F}\times \mathbf{P}\mathbf{C},$$

${\varvec{P}}{\varvec{C}}$ is an M $\times$ N matrix of principal components. **EOF** is the M × M orthogonal matrix. The calculation process is as follows: the original matrix is subject to anomaly processing to obtain a data matrix $\mathbf{X}$, before calculating the multiplication of $\mathrm{X}$ and its transposed matrix ${\mathbf{X}}^{{\varvec{T}}}$ to obtain a square matrix:

$$\mathbf{C}=\frac{1}{\mathbf{N}}\mathbf{X}\times {\mathbf{X}}^{\mathbf{T}},$$

where $\mathbf{X}$ has experienced anomaly treatment and **C** is the covariance matrix. We calculated the eigenvalues (${\lambda }_{1},\cdots ,{\lambda }_{m}$) and eigenvectors **V** of the square matrix **C**, using the following formula:

$${\varvec{C}}\times {\varvec{V}}=\mathbf{V}\times \mathbf{E},$$

where **E** is the diagonal array:

$${\varvec{E}}=\left[\begin{array}{ccc}{\lambda }_{1}& \cdots & 0\\ \vdots & \ddots & \vdots \\ 0& \cdots & {\lambda }_{M}\end{array}\right].$$

In general, eigenvalues are arranged in descending order:${\lambda }_{1}>{\lambda }_{2}>\cdots {>\lambda }_{M}$. Because $\mathrm{X}$ is a real observation, $\uplambda$ is greater than or equal to 0; therefore, each nonzero eigenvalue corresponds to a column of eigenvectors, also known as an EOF. For example, the eigenvectors corresponding to ${\uplambda }_{1}$ are called the first EOF pattern, given by the first column of **V**.

To calculate the PCs, we project the EOFs onto the original data matrix to obtain the time coefficients (PCs) corresponding to all spatial eigenvectors:

$$\mathbf{P}\mathbf{C}={\mathbf{V}}^{\mathbf{T}}\times \mathbf{X},$$

where each line in the PC corresponds to the time coefficients of each eigenvector.

To calculate the variance contribution rate, the variance of matrix $X$ is represented by the measurement of the eigenvalue. The larger the λ, the more important the corresponding EOF and the greater its contribution to the total variance. The contribution of the *k*th pattern to the total variance is: $\frac{{\lambda }_{k}}{{\sum }_{i=1}^{m}{\lambda }_{i}}\times 100\%$. The error bars are given at the 95% confidence level^43^: $\mathrm{\Delta \lambda }=\uplambda \sqrt{2/{N}^{*}}$, where $\uplambda$ is the eigenvalue and ${N}^{*}$ is the effective number of freedom degrees in the data set. We checked the error range of $\uplambda$ in order. If the adjacent two $\uplambda$ ranges overlapped, then the significance test was not passed.
